# A clinically applicable nomogram predicting non-return to work in young and middle-aged patients with acute large vessel occlusion stroke: integrating neurological function and psychosocial factors for personalized rehabilitation

**DOI:** 10.3389/fneur.2026.1837086

**Published:** 2026-06-24

**Authors:** Yu Ding, Jingling Zhu, Yiling He, Xiuling Yang, Wenfei Liang, Kangqiang Yang, Xiaoling Wu, Guoshun Li, Jiasheng Zhao, Zhan Zhao, Jingyi Chen, Qiuxing He, Weimin Ning

**Affiliations:** 1 Department of Neurology, Dongguan Hospital of Guangzhou University of Chinese Medicine, Dongguan, China; 2 Dongguan Key Laboratory of Intractable Brain Diseases, Dongguan Hospital of Guangzhou University of Chinese Medicine, Dongguan, China; 3 State Key Laboratory of Dampness Syndrome of Chinese Medicine, Dongguan Hospital of Guangzhou University of Chinese Medicine, Dongguan, China

**Keywords:** acute ischemic stroke, endovascular therapy, large vessel occlusion, non-return to work, prediction model, vocational outcome

## Abstract

**Objective:**

This study was designed to identify key predictors of non-return to work (non-RTW) in young and middle-aged patients with acute ischemic stroke due to large vessel occlusion (AIS-LVO) after endovascular therapy (EVT). Based on these predictors, we developed and validated an individualized nomogram for non-RTW risk stratification to facilitate early identification of high-risk patients and guide personalized rehabilitation for better functional recovery and less occupational loss.

**Methods:**

In this retrospective cohort study, 350 consecutive AIS-LVO patients who underwent EVT at Dongguan Hospital of Traditional Chinese Medicine (July 2018–July 2025) were included. Potential predictors were selected using least absolute shrinkage and selection operator (LASSO) regression, and independent predictors were identified via multivariable logistic regression. A nomogram was constructed and assessed for discrimination using the area under the receiver operating characteristic curve (AUC), for calibration using calibration curves and the Hosmer–Lemeshow test, and for clinical utility via decision curve analysis (DCA).

**Results:**

Six independent predictors of non-RTW were identified: instrumental activities of daily living (IADL), admission NIHSS score, Nutritional Risk Screening 2002 (NRS-2002) score, balance impairment (as measured by the Berg Balance Scale, BBS), post-stroke rehabilitation (Rehab), and anxiety-depressive state (ADS). The nomogram demonstrated robust discriminative performance (AUC = 0.858, 95% CI: 0.812–0.903). Calibration curves confirmed favorable calibration between predicted and observed probabilities. Decision curve and clinical impact analyses revealed clinically meaningful net benefit across most threshold probabilities.

**Conclusion:**

We developed and validated a clinically actionable nomogram to predict non-RTW in young and middle-aged AIS-LVO patients after EVT. This tool enables early risk stratification and personalized rehabilitation planning, promoting long-term functional and vocational recovery.

## 1 Introduction

Endovascular therapy (EVT) has become a Class I recommended treatment for acute ischemic stroke due to large vessel occlusion (AIS-LVO) (1). Rapid restoration of cerebral blood perfusion by EVT significantly improves recanalization rates and neurological outcomes, and effectively reduces short-term disability and mortality (2). Although EVT provides significant benefits in the acute phase (3, 4), mid-term functional outcomes—such as return to work (RTW)—represent an important indicator for evaluating the overall value of this treatment. Chang WH, Arwert HJ, and others have demonstrated that post-EVT employment status significantly affects patients’ psychological well-being (5), social role identity, and family economic stability. For the young-to-middle-aged stroke cohort, RTW not only reflects functional recovery but also serves as a key marker of therapeutic effectiveness (6).

The present investigation focuses on young and middle-aged stroke patients aged 18–64 years with AIS-LVO, as defined by the Global Burden of Disease (GBD) framework (7). This population represents the primary economic pillar of families, with responsibilities including child-rearing and elder care, and also represents the core component of the labor force. Impairment of work capacity in this group may markedly increase household financial strain and lead to productivity losses (8, 9). Previous studies have shown that young and middle-aged EVT-treated stroke patients have a high rate of non-RTW, and that the incidence of psychological disorders such as depression and anxiety is 1.88–2.75 fold higher in those with non-RTW status compared with those who return to work (10). Beyond income disruption and increased family economic burden, loss of these core workers—combined with reduced social participation due to psychological distress—further increases the demand on social support systems, resulting in multifaceted negative impacts on both families and society (11, 12).

Currently, no universally accepted definition exists of post-stroke RTW in the international literature. Based on existing studies and clinical practice, RTW was defined as re-engagement in remunerated occupational activity, encompassing full- or part-time arrangements, self-employment, standard employment, or modified work roles (13), with an average working time of no less than 10 h per week (14). Previous studies indicate that approximately 40–60% of stroke survivors are non-RTW (15). However, current RTW prediction largely relies on clinicians’ subjective judgment, and objective, quantitative tools remain lacking. Previous research has primarily focused on neurological function, physical disability, and demographic factors (16, 17), without integrating neurological, cognitive, and psychological dimensions into a comprehensive predictive framework. This limitation hinders the timely stratification of high-risk cases and the prompt deployment of multidisciplinary interventions.

Accordingly, this investigation aims to systematically identify factors of non-RTW following EVT in working-age AIS-LVO patients using a retrospective cohort design, and to develop a visual prognostic nomogram. The nomogram is intended to facilitate early clinical screening of high-risk patients and guide the implementation of personalized interventions, including vocational rehabilitation, nutritional support, balance training, and sleep management, ultimately improving post-stroke social and occupational functional outcomes.

## 2 Methods

### 2.1 Study design and participants

This study was a single-center retrospective observational study. Study participants were identified from consecutive patients with acute ischemic stroke who received endovascular therapy at Dongguan Hospital of Traditional Chinese Medicine during the period from July 2018 through July 2025. Data were retrieved via the institutional electronic health record system, an initial search was conducted with the strategy “acute ischemic stroke + endovascular therapy,” yielding 637 potentially eligible cases. These cases were subsequently screened rigorously according to predefined eligibility criteria.

#### 2.1.1 Inclusion criteria

(1) Acute ischemic stroke (AIS) diagnosis conforming to the diagnostic criteria specified in the *Guidelines for the Diagnosis and Treatment of Acute Ischemic Stroke*, with the presence of intracranial large vessel occlusion (LVO) confirmed by computed tomography angiography (CTA), magnetic resonance angiography (MRA), or digital subtraction angiography (DSA). LVO was operationalized as occlusion involving the intracranial internal carotid artery, M1 middle cerebral artery, A1 anterior cerebral artery, or basilar artery. (2) Age between 18 and 64 years, consistent with the definition of the young and middle-aged population (18); (3) A stable occupational status within the 6 months preceding stroke onset, defined as working ≥10 h per week, encompassing full- versus part-time occupational engagement, and self-employment modalities. Individuals engaged only in temporary work, household duties, student status, or unemployed/job-seeking status were excluded.

#### 2.1.2 Exclusion criteria

(1) A prior diagnosis of severe cognitive impairment or psychiatric disorders; (2) Unemployed, retired, or economically inactive status prior to stroke onset; (3) Presence of other severe conditions that could substantially affect functional recovery, including serious orthopedic diseases, malignancies, or severe cardiac, hepatic, or renal failure; (4) Missing key clinical data in the medical records; (5) Failure to complete follow-up, including refusal of follow-up by the patient or family members, or loss to follow-up due to invalid contact information.

### 2.2 Participant selection results

According to the aforementioned criteria, a total of 287 patients were excluded for the following reasons: presence of severe comorbid conditions (*n* = 79), lack of stable employment prior to stroke (*n* = 63), incomplete clinical data (*n* = 82), and loss to follow-up (*n* = 63). Ultimately, the analytical cohort comprised 350 patients.

### 2.3 Follow-up and outcome definition

All patients completed a 90-day post-procedural follow-up. Follow-up assessments were conducted either through outpatient visits or structured telephone interviews. For patients residing locally or with adequate mobility, face-to-face evaluations were performed by specially trained healthcare professionals. For those living outside the region or with limited mobility, standardized telephone interviews were used.

RTW status at the 90-day interval following EVT constituted the primary endpoint. RTW was operationally defined as resumption of the pre-stroke occupation or a job requiring comparable functional skills, with work intensity and weekly working hours reaching at least 70% of the pre-stroke level. RTW status was recorded as a binary outcome (yes/no).

### 2.4 Definition of return to work

The 10-h/week minimum was selected based on established international definitions of RTW after stroke, which recognize part-time work (> = 25% of full-time, or ~10 h/week) as valid vocational reintegration (19, 20). We acknowledge that this threshold is debated in the literature; however, for AIS-LVO patients with severe initial neurological deficits, sustained part-time employment represents meaningful functional recovery and preserves occupational identity, which are patient-centered outcomes of clinical significance (13).

Post-stroke RTW definitions vary substantially, with thresholds ranging from ≥1 h/week to full-time employment. We defined functional RTW as resumption of ≥70% pre-stroke work intensity to distinguish “functional return to work” from “symbolic return to work”. This threshold is relatively stringent: Radford et al. reported only one-third of full-time stroke survivors resumed full-time work at 12 months (21). For young and middle-aged AIS-LVO patients undergoing EVT, this threshold reflects clinically meaningful recovery while acknowledging that complete restoration may require extended rehabilitation.

The 90-day endpoint aligns with the plateau of spontaneous neurological recovery and AHA/ASA guidelines for functional assessment. In our single-center Chinese setting—where hospitalization is brief, community rehabilitation is limited, and longer follow-up faces retention challenges—this window is clinically feasible and consistent with post-EVT protocols. Although Radford et al. reported a mean RTW time of 90 days (21), we acknowledge that RTW rates increase over longer intervals; our outcome therefore reflects early post-EVT recovery rather than long-term trajectories.

### 2.5 Clinical data collection and variable definitions

Informed by a systematic literature review and clinical expertise, 27 potential predictive variables were initially identified. Data acquisition via the institutional electronic health record platform encompassed the following variable domains:

*Baseline variables*: demographics (age, sex, marital status, income level); vascular risk factors (hypertension, diabetes, coronary disease, smoking);stroke etiology (TOAST classification); National Institutes of Health Stroke Scale (NIHSS; range, 0–42, with higher scores indicating greater neurological deficit. mild <5, moderate 5–15, severe >16); Glasgow Coma Scale (GCS; total score 15; mild injury 13–15, moderate injury 9–12, severe injury 3–8); modified Rankin Scale (mRS; total score 6; favorable outcome 0–2, unfavorable outcome 3–6) (22); Instrumental Activities of Daily Living (IADL) assessed using the Lawton IADL scale (total score 0–8 for women / 0–7 for men; fully independent: 8 [women]/7 [men]; largely independent: 6–7 [women]/5–6 [men]; partially dependent: 3–5 [women]/3–4 [men]; fully dependent: 0–2 [women]/0–2 [men]) (23), and the Berg Balance Scale (BBS; total score 56; high fall risk 0–20, moderate fall risk 21–44, low fall risk ≥45) (24); Anxiety-Depressive State (ADS, assessed using the Hamilton Anxiety Scale [HAMA] and Hamilton Depression Scale [HAMD], with final results presented as a binary classification indicating the presence or absence of anxiety-depressive state); sleep quality (Pittsburgh Sleep Quality Index [PSQI]); nutritional risk (Nutritional Risk Screening 2002 [NRS-2002] ≥ 3); and neurological symptoms (dysarthria, sensory deficits).

*Procedural variable*: EVT duration (groin puncture to reperfusion).

*Postoperative variables*: glycated hemoglobin (HbA1c), serum creatinine, left ventricular ejection fraction (LVEF), Fazekas grade, prognostic nutritional index (PNI), and atherosclerosis index (AI).

*Treatment and interventions*: receipt of standardized post-stroke rehabilitation therapy, defined as early initiation of multidisciplinary comprehensive rehabilitation after stabilization of vital signs, in accordance with the U. S. Department of Defense Clinical Practice Guidelines (25).

All functional assessments were performed by three trained neurologists (inter-rater *κ* ≥ 0.75), with two independent raters and a third adjudicator for discrepancies.

### 2.6 Statistical analysis

All statistical analyses and graphical visualizations were performed using SPSS 27.0, R 4.2.3, and Origin 2024 software.

#### 2.6.1 Data preprocessing

Missing data rates for all candidate predictors are presented in Supplementary Table S1, with missingness ranging from 0.0 to 5.7%. Variables with <10% missing data were handled using multiple imputation via the Fully Conditional Specification (FCS) method implemented in the SPSS Missing Values module. Ten imputed datasets were generated and pooled using Rubin’s rules. Sensitivity analyses yielded results consistent with complete-case analyses, supporting the robustness of the imputation approach. Multicollinearity among 27 potential predictors was also assessed; all variance inflation factors (VIFs) were <5, suggesting no significant multicollinearity and permitting inclusion of all variables in subsequent modeling.

#### 2.6.2 Statistical methods

① Continuous variables were tested for normality using the Shapiro–Wilk test. Normally distributed data were expressed as mean ± standard deviation and compared using independent-samples t-tests; non-normally distributed data were presented as median (interquartile range) and compared using Mann–Whitney U tests. ② Categorical variables were expressed as percentages and compared using χ^2^ or Fisher’s exact tests, as appropriate. ③ Variable selection and model construction: Least Absolute Shrinkage and Selection Operator (LASSO) regression was used to identify significant predictors (*p* < 0.05), which were entered into multivariable Logistic regression to calculate odds ratios (ORs) and construct the predictive model. A nomogram was developed for visualization.

#### 2.6.3 Model validation

The predictive performance of the nomogram was evaluated using receiver operating characteristic (ROC) curve analysis; calibration was assessed using calibration curves; and clinical utility was examined through decision curve analysis (DCA).

#### 2.6.4 Temporal sensitivity analysis

To assess whether evolving EVT techniques, post-procedural care protocols, and rehabilitation paradigms over the 7-year study period introduced temporal confounding, we performed two supplementary analyses. First, the cohort was divided into three eras (2018–2020, 2021–2023, and 2024–2025), and multivariable logistic regression models were fitted within each stratum to compare the stability of odds ratios and discriminative performance (AUC). Second, calendar year was entered as a continuous covariate in the main multivariable model to evaluate era effects.

To evaluate potential confounding by indication, a propensity score matching sensitivity analysis was performed. Propensity scores were estimated using a logistic regression model incorporating age, sex, admission NIHSS, admission GCS, admission mRS, IADL, BBS, ADS, NRS-2002, dysarthria, hypertension, and diabetes mellitus. Patients receiving rehabilitation were matched 1:1 to controls using nearest-neighbor matching with a caliper of 0.02. Covariate balance was assessed via propensity score distributions and standardized mean differences (SMDs); SMD < 0.10 indicated adequate balance, and <0.20 acceptable. The average treatment effect on the treated (ATT) was subsequently estimated.

## 3 Results

### 3.1 Study flow chart

**Figure 1:**
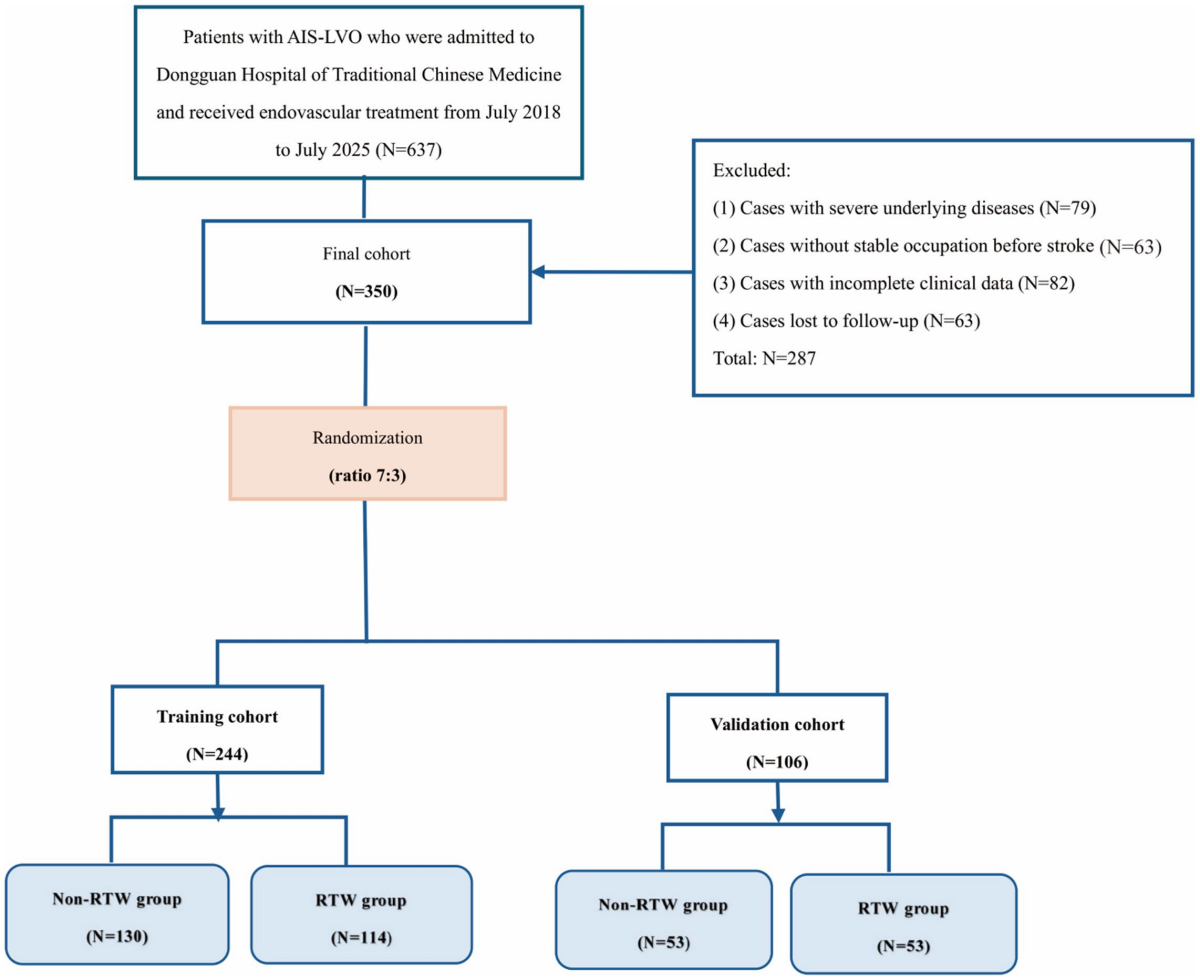
Flow chart of the data selection process. The chart illustrates the patient selection process for young and middle-aged adults with AIS-LVO undergoing EVT. Detailed screening criteria and sample size at each stage (screened, excluded, and finally included) are presented, with reasons for exclusion specified.

Figure 1 presents the patient selection workflow. From an initial screening of 637 patients, 287 were excluded based on predefined eligibility criteria, yielding a final analytical cohort of 350 patients.

### 3.2 Baseline characteristics of the patients

The total cohort of 350 patients was randomly partitioned into a training cohort (*n* = 244) and a validation cohort (*n* = 106) at a ratio of 7:3. Baseline characteristic comparisons revealed no statistically significant differences between the two cohorts across all variables (all *p* > 0.05). The baseline characteristics of the two cohorts are summarized and compared in Table 1, demonstrating a balanced distribution of baseline variables between the training and validation cohorts.

**Table 1:** Comparison of baseline characteristics between the training and validation cohorts.

Variables	Total (*n* = 350)	Validation Cohort (*n* = 106)	Training Cohort (*n* = 244)	Statistic	*p*
Outcome, *n* (%)				χ^2^ = 0.318	0.573
RTW Group	183 (52.29)	53 (50.00)	130 (53.28)		
Non-RTW Group	167 (47.71)	53 (50.00)	114 (46.72)		
Age, M (Q₁, Q₃)	55.00 (48.00,60.00)	55.00 (48.00,61.00)	56.00 (48.00,60.00)	Z = 0.410	0.682
Surgery, M (Q₁, Q₃)	1.46 (0.96,1.94)	1.38 (0.96,1.93)	1.46 (0.99,1.95)	Z = -0.807	0.420
HbA1c, M (Q₁, Q₃)	5.90 (5.50,6.79)	5.90 (5.50,6.97)	5.80 (5.50,6.70)	Z = 0.494	0.621
PNI, M (Q₁, Q₃)	48.20 (44.60,52.90)	48.20 (44.56,53.44)	48.20 (44.60,52.15)	Z = 0.249	0.803
AI, M (Q₁, Q₃)	3.53 (2.57,4.27)	3.34 (2.48,4.05)	3.58 (2.63,4.34)	Z = -1.157	0.247
LVEF, M (Q₁, Q₃)	67.00 (62.97,72.00)	65.00 (61.12,71.39)	67.00 (63.00,72.00)	Z = -1.591	0.112
SCr, M (Q₁, Q₃)	75.05 (63.47,92.30)	72.35 (60.35,94.92)	75.80 (64.00,91.00)	Z = -0.277	0.782
Gender, *n* (%)				χ^2^ = 1.003	0.317
Female	68 (19.43)	24 (22.64)	44 (18.03)		
Male	282 (80.57)	82 (77.36)	200 (81.97)		
Hypertension, *n* (%)				χ^2^ = 0.128	0.720
None	147 (42.00)	43 (40.57)	104 (42.62)		
Yes	203 (58.00)	63 (59.43)	140 (57.38)		
Diabetes, *n* (%)				χ^2^ = 0.486	0.486
None	266 (76.00)	78 (73.58)	188 (77.05)		
Yes	84 (24.00)	28 (26.42)	56 (22.95)		
CVD, *n* (%)				χ^2^ = 0.056	0.813
None	288 (82.29)	88 (83.02)	200 (81.97)		
Yes	62 (17.71)	18 (16.98)	44 (18.03)		
Smoking, *n* (%)				χ^2^ = 2.616	0.106
None	201 (57.43)	54 (50.94)	147 (60.25)		
Yes	149 (42.57)	52 (49.06)	97 (39.75)		
ADS, *n* (%)				χ^2^ = 0.033	0.856
None	302 (86.29)	92 (86.79)	210 (86.07)		
Yes	48 (13.71)	14 (13.21)	34 (13.93)		
Sleep disorder, *n* (%)				χ^2^ = 0.036	0.849
None	273 (78.00)	82 (77.36)	191 (78.28)		
Yes	77 (22.00)	24 (22.64)	53 (21.72)		
Marriage, *n* (%)				χ^2^ = 0.236	0.627
Unmarried	23 (6.57)	8 (7.55)	15 (6.15)		
Married	327 (93.43)	98 (92.45)	229 (93.85)		
Dysarthria, *n* (%)				χ^2^ = 0.592	0.442
None	75 (21.43)	20 (18.87)	55 (22.54)		
Yes	275 (78.57)	86 (81.13)	189 (77.46)		
Admission mRS, *n* (%)				χ^2^ = 1.853	0.173
Score 0–2	100 (28.57)	25 (23.58)	75 (30.74)		
Score 3–6	250 (71.43)	81 (76.42)	169 (69.26)		
Rehab, *n* (%)				χ^2^ = 0.213	0.644
None	116 (33.14)	37 (34.91)	79 (32.38)		
Yes	234 (66.86)	69 (65.09)	165 (67.62)		
Paresthesia, *n* (%)				χ^2^ = 0.041	0.839
None	245 (70.00)	75 (70.75)	170 (69.67)		
Paresthesia	105 (30.00)	31 (29.25)	74 (30.33)		
NRS-2002, *n* (%)				χ^2^ = 1.409	0.235
None	287 (82.00)	83 (78.30)	204 (83.61)		
Yes	63 (18.00)	23 (21.70)	40 (16.39)		
Admission GCS, *n* (%)				χ^2^ = 1.282	0.527
Score 13–15	279 (79.71)	83 (78.30)	196 (80.33)		
Score 9–12	49 (14.00)	14 (13.21)	35 (14.34)		
Score 3–8	22 (6.29)	9 (8.49)	13 (5.33)		
Admission NIHSS, *n* (%)				χ^2^ = 2.677	0.262
Score <5	98 (28.00)	26 (24.53)	72 (29.51)		
Score 5–15	207 (59.14)	62 (58.49)	145 (59.43)		
Score >16	45 (12.86)	18 (16.98)	27 (11.07)		
Income, *n* (%)				χ^2^ = 0.349	0.986
Low	38 (10.85)	12 (11.32)	26 (10.66)		
Medium	246 (70.28)	76 (71.70)	170 (69.67)		
High	66 (18.87)	18 (16.98)	48 (19.67)		
TOAST, *n* (%)				χ^2^ = 2.768	0.597
LAA	263 (75.14)	81 (76.42)	182 (74.59)		
CE	38 (10.86)	12 (11.32)	26 (10.66)		
SVO	9 (2.57)	1 (0.94)	8 (3.28)		
SOE	25 (7.14)	9 (8.49)	16 (6.56)		
UE	15 (4.29)	3 (2.83)	12 (4.92)		
Fazekas, *n* (%)				χ^2^ = 0.669	0.880
No WMH	148 (42.29)	45 (42.45)	103 (42.21)		
Punctate foci	141 (40.29)	45 (42.45)	96 (39.34)		
Beginning confluence of foci	45 (12.86)	12 (11.32)	33 (13.52)		
Large confluent areas	16 (4.57)	4 (3.77)	12 (4.92)		
BBS, *n* (%)				χ^2^ = 1.378	0.502
Low	172 (49.14)	50 (47.17)	122 (50.00)		
Medium	124 (35.43)	36 (33.96)	88 (36.07)		
High	54 (15.43)	20 (18.87)	34 (13.93)		
IADL, *n* (%)				χ^2^ = 1.040	0.791
Independent	61 (17.43)	17 (16.04)	44 (18.03)		
Mild dependence	126 (36.00)	36 (33.96)	90 (36.89)		
Moderate dependence	85 (24.29)	26 (24.53)	59 (24.18)		
Severe dependence	78 (22.29)	27 (25.47)	51 (20.90)		

HbA1c, glycated hemoglobin; PNI, prognostic nutritional index calculated as 10 × serum albumin + 5 × lymphocyte count;ity lipoprotein cholesterol; AI, atherosclerosis index calculated as (total cholesterol − high-density lipoprotein cholesterol)/high-density lipoprotein cholesterol; EF, left ventricular ejection fraction; SCr, serum creatinine; CVD, cardiovascular disease; ADS, Anxiety-Depressive State; mRS, modified Rankin Scale; NRS-2002, Nutritional Risk Screening 2002; GCS, Glasgow Coma Scale; NIHSS, National Institutes of Health Stroke Scale; LAA, large-artery atherosclerosis; CE, cardioembolism; SVO, small-vessel occlusion; SOE, stroke of other determined etiology; UE, undetermined etiology; WMH, white matter hyperintensity; BBS, Berg Balance Scale; Lawton-IADL, Lawton Instrumental Activities of Daily Living scale.

### 3.3 Comparative analysis of the training cohort

In the comparative analysis of the training cohort, statistically significant differences were observed between the non-RTW group (*n* = 130) and the RTW group (*n* = 114) in the following variables: Admission GCS, Admission NIHSS, Admission mRS, IADL, dysarthria, anxiety-depressive state (ADS), rehabilitation, BBS, NRS-2002, (all *p* < 0.05). No statistically significant differences were found in age, surgery, HbA1c, PNI, AI, LVEF, SCr, gender, hypertension, diabetes, CVD, smoking, sleep disorder, marriage, paresthesia, income, TOAST, Fazekas (all *p* > 0.05). The results are presented in Table 2.

**Table 2:** Comparative analysis of baseline characteristics in the training cohort.

Variables	Total (*n* = 244)	non-RTW group (*n* = 130)	RTW group (*n* = 114)	Statistic	*p*
Age, M (Q₁, Q₃)	56.00 (48.00,60.00)	55.00 (49.00,60.00)	57.00 (47.25,61.00)	Z = -0.621	0.535
Surgery, M (Q₁, Q₃)	1.46 (0.99,1.95)	1.38 (1.01,1.91)	1.55 (0.98,2.08)	Z = -0.931	0.352
HbA1c, M (Q₁, Q₃)	5.80 (5.50,6.70)	5.80 (5.40,6.63)	5.87 (5.60,6.99)	Z = -1.192	0.233
PNI, M (Q₁, Q₃)	48.20 (44.60,52.15)	49.05 (44.73,53.50)	47.33 (44.60,50.52)	Z = 1.622	0.105
AI, M (Q₁, Q₃)	3.58 (2.63,4.34)	3.57 (2.58,4.46)	3.60 (2.68,4.20)	Z = 0.139	0.889
LVEF, M (Q₁, Q₃)	67.00 (63.00,72.00)	67.00 (63.00,71.00)	68.00 (63.00,73.00)	Z = -0.747	0.455
SCr, M (Q₁, Q₃)	75.80 (64.00,91.00)	75.05 (64.00,88.38)	75.95 (64.00,94.97)	Z = -0.467	0.640
Gender, *n* (%)				χ^2^ = 2.198	0.138
Female	44 (18.03)	19 (14.62)	25 (21.93)		
Male	200 (81.97)	111 (85.38)	89 (78.07)		
Hypertension, *n* (%)				χ^2^ = 1.970	0.160
None	104 (42.62)	50 (38.46)	54 (47.37)		
Yes	140 (57.38)	80 (61.54)	60 (52.63)		
Diabetes, *n* (%)				χ^2^ = 0.749	0.387
None	188 (77.05)	103 (79.23)	85 (74.56)		
Yes	56 (22.95)	27 (20.77)	29 (25.44)		
CVD, *n* (%)				χ^2^ = 1.320	0.251
None	200 (81.97)	110 (84.62)	90 (78.95)		
Yes	44 (18.03)	20 (15.38)	24 (21.05)		
Smoking, *n* (%)				χ^2^ = 1.283	0.257
None	147 (60.25)	74 (56.92)	73 (64.04)		
Yes	97 (39.75)	56 (43.08)	41 (35.96)		
ADS, *n* (%)				χ^2^ = 20.149	**<0.001**
None	210 (86.07)	124 (95.38)	86 (75.44)		
Yes	34 (13.93)	6 (4.62)	28 (24.56)		
Sleep Disorder, *n* (%)				χ^2^ = 5.072	0.024
None	191 (78.28)	109 (83.85)	82 (71.93)		
Yes	53 (21.72)	21 (16.15)	32 (28.07)		
Marriage, *n* (%)				χ^2^ = 0.290	0.590
Unmarried	15 (6.15)	9 (6.92)	6 (5.26)		
Married	229 (93.85)	121 (93.08)	108 (94.74)		
Dysarthria, *n* (%)				χ^2^ = 20.367	**<0.001**
None	55 (22.54)	44 (33.85)	11 (9.65)		
Yes	189 (77.46)	86 (66.15)	103 (90.35)		
Admission mRS, *n* (%)				χ^2^ = 31.061	**<0.001**
Score 0–2	75 (30.74)	60 (46.15)	15 (13.16)		
Score 3–6	169 (69.26)	70 (53.85)	99 (86.84)		
Rehab, *n* (%)				χ^2^ = 21.964	**<0.001**
None	79 (32.38)	25 (19.23)	54 (47.37)		
Yes	165 (67.62)	105 (80.77)	60 (52.63)		
Paresthesia, *n* (%)				χ^2^ = 2.294	0.130
None	170 (69.67)	96 (73.85)	74 (64.91)		
Paresthesia	74 (30.33)	34 (26.15)	40 (35.09)		
NRS-2002, *n* (%)				χ^2^ = 15.370	**<0.001**
None	204 (83.61)	120 (92.31)	84 (73.68)		
Yes	40 (16.39)	10 (7.69)	30 (26.32)		
Admission GCS, *n* (%)				χ^2^ = 19.223	**<0.001**
Score 13–15	196 (80.33)	118 (90.77)	78 (68.42)		
Score 9–12	35 (14.34)	9 (6.92)	26 (22.81)		
Score 3–8	13 (5.33)	3 (2.31)	10 (8.77)		
Admission NIHSS, *n* (%)				χ^2^ = 54.626	**<0.001**
Score <5	72 (29.51)	62 (47.69)	10 (8.77)		
Score 5–15	145 (59.43)	65 (50.00)	80 (70.18)		
Score >16	27 (11.07)	3 (2.31)	24 (21.05)		
Income, *n* (%)				χ^2^ = 0.798	0.939
Low	26 (10.66)	16 (12.31)	10 (8.77)		
Medium	170 (69.67)	89 (68.46)	81 (71.05)		
High	48 (19.67)	25 (19.23)	23 (20.18)		
TOAST, *n* (%)				χ^2^ = 2.691	0.611
LAA	182 (74.59)	100 (76.92)	82 (71.93)		
CE	26 (10.66)	11 (8.46)	15 (13.16)		
SVO	8 (3.28)	4 (3.08)	4 (3.51)		
SOE	16 (6.56)	10 (7.69)	6 (5.26)		
UE	12 (4.92)	5 (3.85)	7 (6.14)		
Fazekas, *n* (%)				χ^2^ = 4.183	0.242
No WMH	103 (42.21)	48 (36.92)	55 (48.25)		
Punctate foci	96 (39.34)	57 (43.85)	39 (34.21)		
Beginning confluence of foci	33 (13.52)	17 (13.08)	16 (14.04)		
Large confluent areas	12 (4.92)	8 (6.15)	4 (3.51)		
BBS, *n* (%)				χ^2^ = 13.127	**0.001**
Low	122 (50.00)	78 (60.00)	44 (38.60)		
Medium	88 (36.07)	41 (31.54)	47 (41.23)		
High	34 (13.93)	11 (8.46)	23 (20.18)		
IADL, *n* (%)				χ^2^ = 39.622	**<0.001**
Independent	44 (18.03)	38 (29.23)	6 (5.26)		
Mild dependence	90 (36.89)	54 (41.54)	36 (31.58)		
Moderate dependence	59 (24.18)	25 (19.23)	34 (29.82)		
Severe dependence	51 (20.90)	13 (10.00)	38 (33.33)		

Bold values indicate test statistics and *p*-values for baseline comparisons between training and validation cohorts. Mann-Whitney U test (Z) for continuous variables; χ² test for categorical variables. All *p* > 0.05, suggesting well-balanced baseline characteristics.

### 3.4 Selection of predictive factors

**Figure 2:**
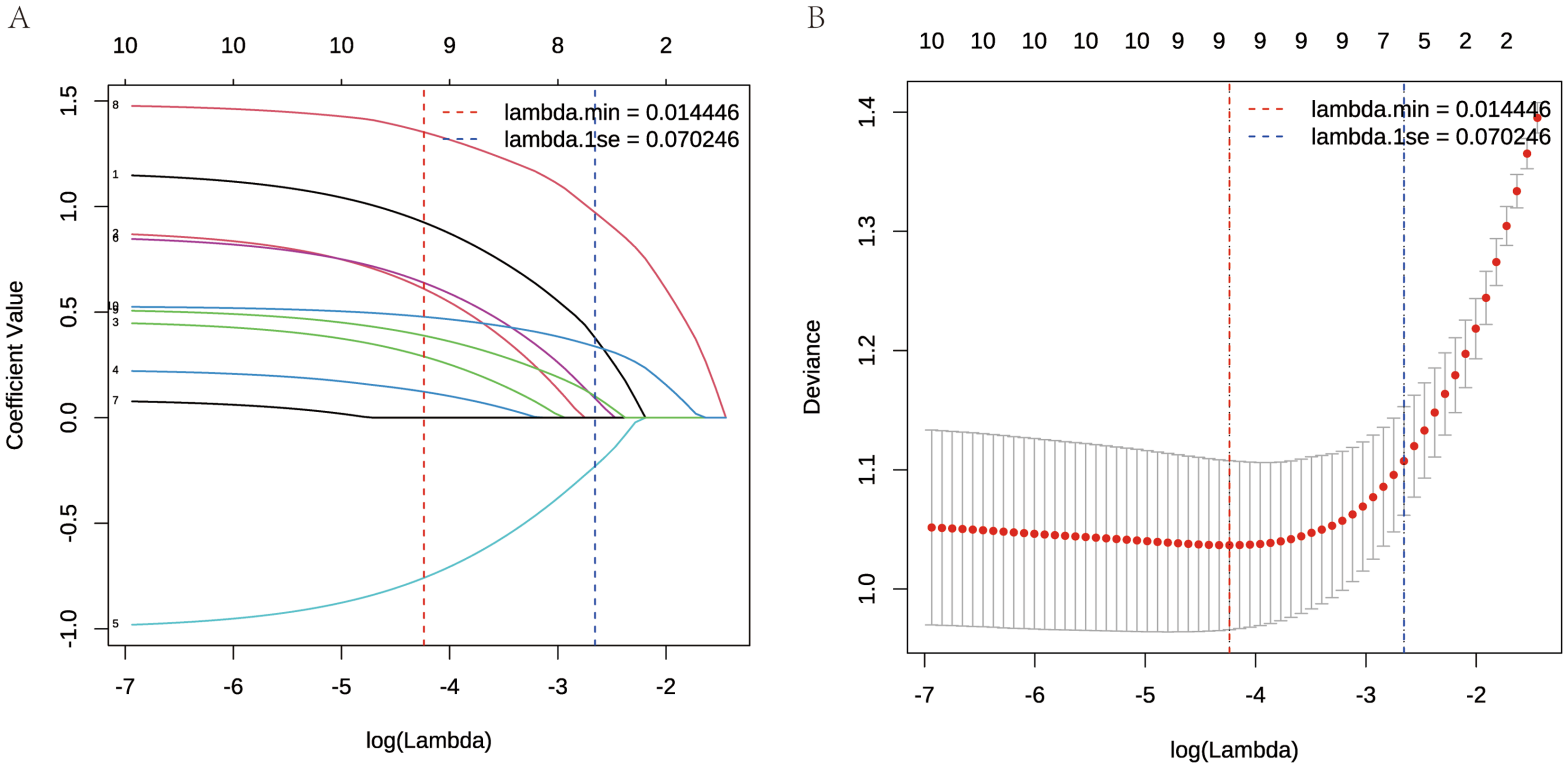
LASSO regression for variable selection. **(A)** Coefficient paths of 27 candidate variables plotted against log(*λ*). The vertical dashed line indicates the optimal tuning parameter (λ = 0.070) selected using the 1-standard-error rule, retaining 6 variables with non-zero coefficients. **(B)** Binomial deviance plot: Mean deviance (*y*-axis) vs. log(λ) (*x*-axis); vertical dashed line marks λ₁se (λ = 0.070) from 10-fold cross-validation, which balances model fit and parsimony.

LASSO regression facilitated screening of 27 candidate predictors, with the optimal regularization coefficient (lambda.1se = 0.070) established through cross-validation, and six predictors with non-zero coefficients were ultimately identified: IADL, Admission NIHSS score, nutritional risk assessed by NRS-2002, balance impairment evaluated using the BBS, post-stroke rehabilitation therapy (Rehab), and ADS. The results of the variable selection process are shown in Figures 2A,B.

**Figure 3:**
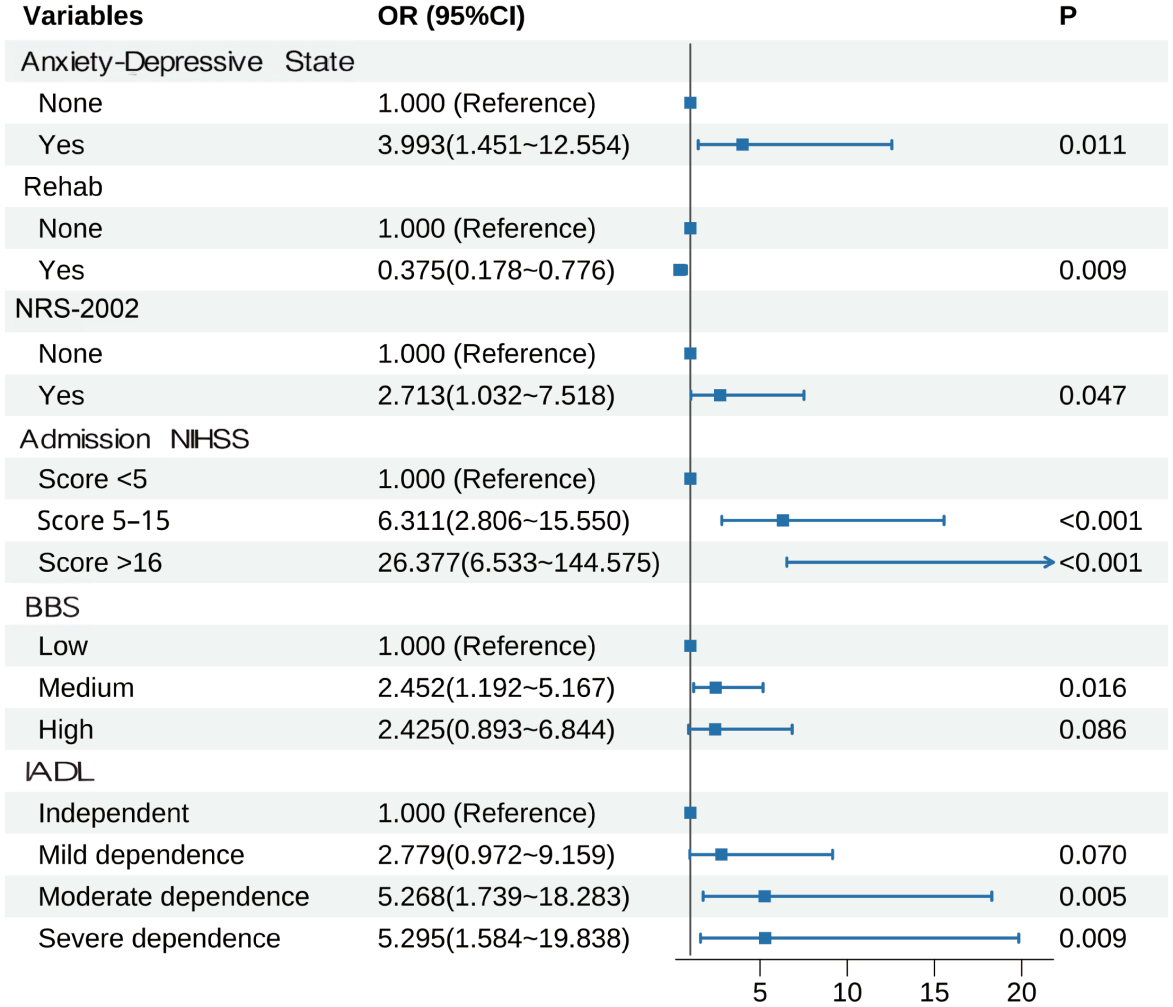
Forest plot of multivariable logistic regression analysis for non-RTW prediction.

Multivariable Logistic regression analysis was performed on the six selected variables. The results indicated that ADS (OR = 3.993, *p* = 0.011), NRS-2002 score (OR = 2.713, *p* = 0.047), and elevated admission NIHSS score (5–15 points: OR = 6.311; >16 points: OR = 26.377, both *p* < 0.001) were independent risk factors for non-RTW following EVT in AIS-LVO patients, whereas rehabilitation therapy (OR = 0.375, *p* = 0.009) served as an independent protective factor. Patients with moderate balance impairment (BBS score 21–44; OR = 2.452, *p* = 0.016) had a significantly increased risk of unsuccessful return to work. A similar trend was observed in patients with severe balance impairment (BBS score 0–20; OR = 2.425, *p* = 0.086), although the association did not reach statistical significance. Moderate-to-severe IADL dependency was also associated with a higher risk of non-RTW (moderate: OR = 5.268, *p* = 0.009; severe: OR = 5.295, *p* = 0.005) (Figure 3). Collinearity diagnostics revealed that variance inflation factors (VIF) for all included variables ranged from 1.039 to 1.147 (all < 5), with tolerances ranging from 0.872 to 0.962 (all > 0.2), indicating no significant multicollinearity among variables and satisfying the statistical requirements for model construction (Table 3).

**Table 3:** Collinearity diagnostics.

Variables	Tolerance value	VIF
ADS	0.962	1.039
Rehab	0.907	1.103
NRS-2002	0.886	1.129
Admission NIHSS	0.931	1.075
BBS	0.872	1.147
IADL	0.895	1.117

### 3.5 Construction of the nomogram

**Figure 4:**
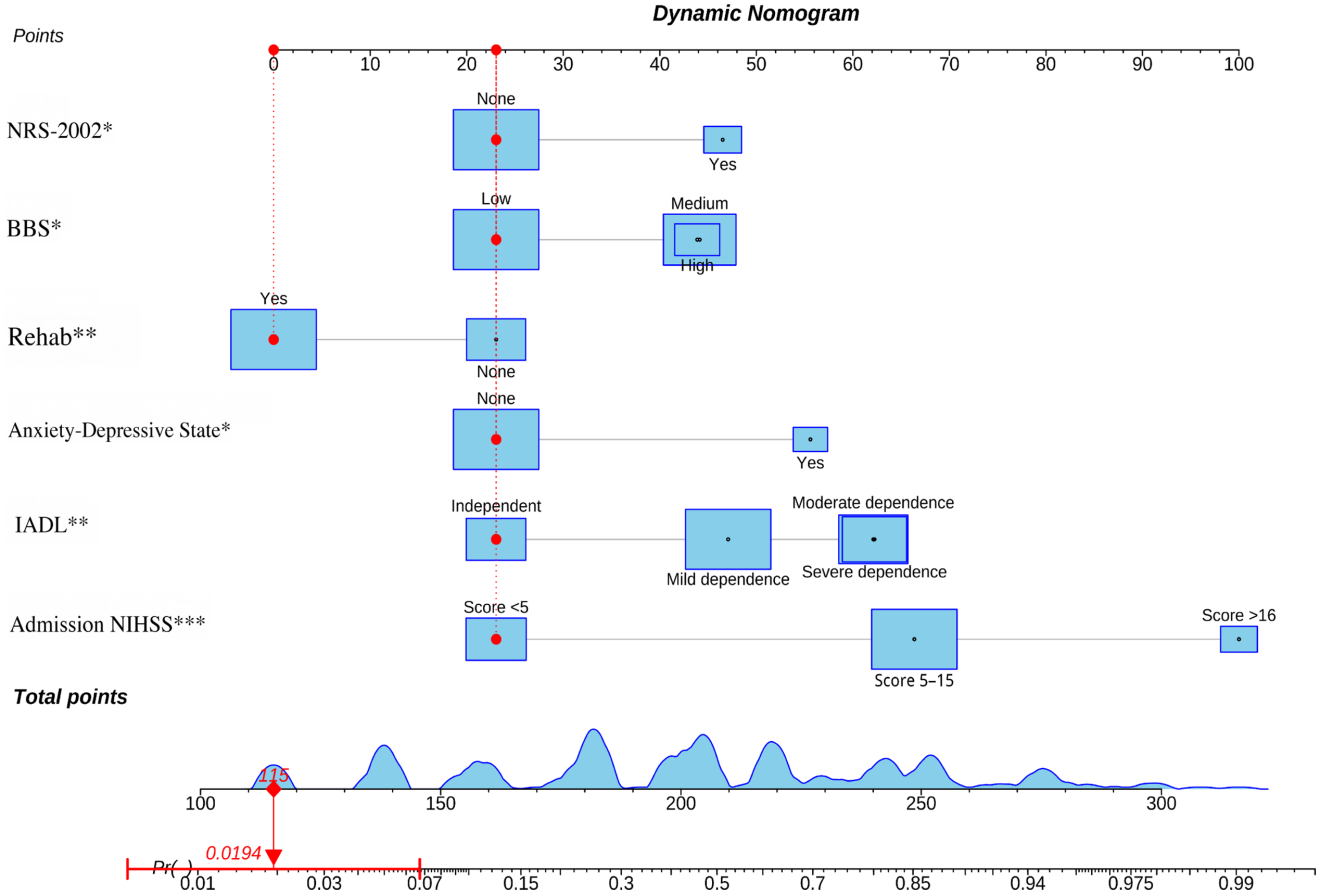
Nomogram for predicting the risk of non-return-to-work (non-RTW) after EVT in patients with AIS-LVO. For each predictor, a corresponding score is assigned according to its category. The Total Points value is obtained by summing the scores of all predictors and is subsequently mapped to the predicted probability of non-RTW. Higher total scores indicate a greater predicted risk of non-RTW. A worked example demonstrating nomogram application is provided in Supplementary Figure S1.

Informed by multivariable logistic regression analysis, a prognostic nomogram was constructed to estimate post-EVT non-RTW risk (Figure 4). Each predictor contributes a weighted point value according to its category, and the total score is calculated by summing the individual predictor scores. The corresponding probability of non-RTW is then obtained by projecting the Total Points value onto the probability scale. To facilitate clinical interpretation and application, a worked example is provided in Supplementary Figure S1.

### 3.6 ROC validation of the predictive model

**Figure 5:**
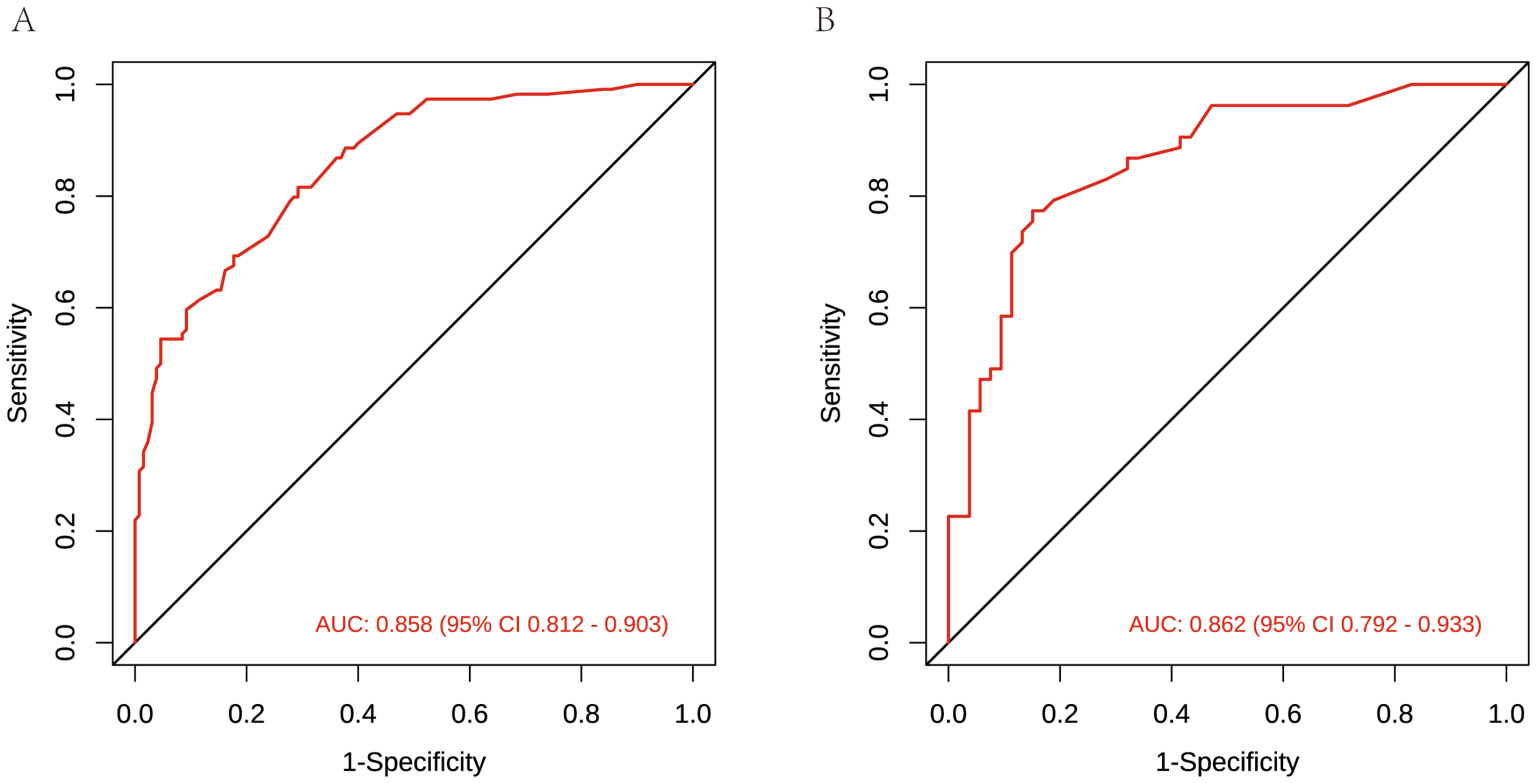
ROC curves demonstrate the performance of the non-RTW predictive nomogram in AIS-LVO patients after EVT. The training cohort **(A)** achieved an AUROC of 0.858 (95% CI: 0.812–0.903), indicating good discriminative ability. In the validation cohort **(B)**, the AUROC was 0.862 (95% CI: 0.792–0.933), further confirming the model’s stable performance in distinguishing patients with and without non-RTW.

As shown in Table 4, to comprehensively assess the model’s discriminatory and diagnostic performance, we report key metrics including the area under the receiver operating characteristic curve (AUC), sensitivity (SEN), specificity (SPE), positive predictive value (PPV), and negative predictive value (NPV) [with 95% confidence intervals (CIs)]. As summarized in Table 4, the model achieved an AUC of 0.858 (training set, 95% CI: 0.812–0.903) and 0.862 (validation set, 95% CI: 0.792–0.933), indicating favorable discriminatory ability. For the training set, the corresponding metrics (with 95% CIs) were SEN = 0.816 (0.745–0.887), SPE = 0.708 (0.63–0.786), PPV = 0.71 (0.632–0.788), and NPV = 0.814 (0.742–0.886); the validation set exhibited SEN = 0.774 (0.661–0.886), SPE = 0.849 (0.753–0.945), PPV = 0.837 (0.733–0.944), and NPV = 0.789 (0.684–0.895). These results collectively demonstrate the model’s consistent diagnostic performance across both cohorts (Figure 5).

**Table 4:** Diagnostic performance indicators of the model based on preset clinical decision thresholds.

Indicator	Training cohort (95% CI)	Validation cohort (95% CI)
AUC	0.858 (0.812 ~ 0.903)	0.862 (0.792 ~ 0.933)
SEN	0.816 (0.745 ~ 0.887)	0.774 (0.661 ~ 0.886)
SPE	0.708 (0.630 ~ 0.786)	0.849 (0.753 ~ 0.945)
PPV	0.710 (0.632 ~ 0.788)	0.837 (0.733 ~ 0.944)
NPV	0.814 (0.742 ~ 0.886)	0.789 (0.684 ~ 0.895)

CI, Confidence interval; PPV, Positive predictive value; NPV, Negative predictive value.

### 3.7 Model performance and clinical value validation

**Figure 6:**
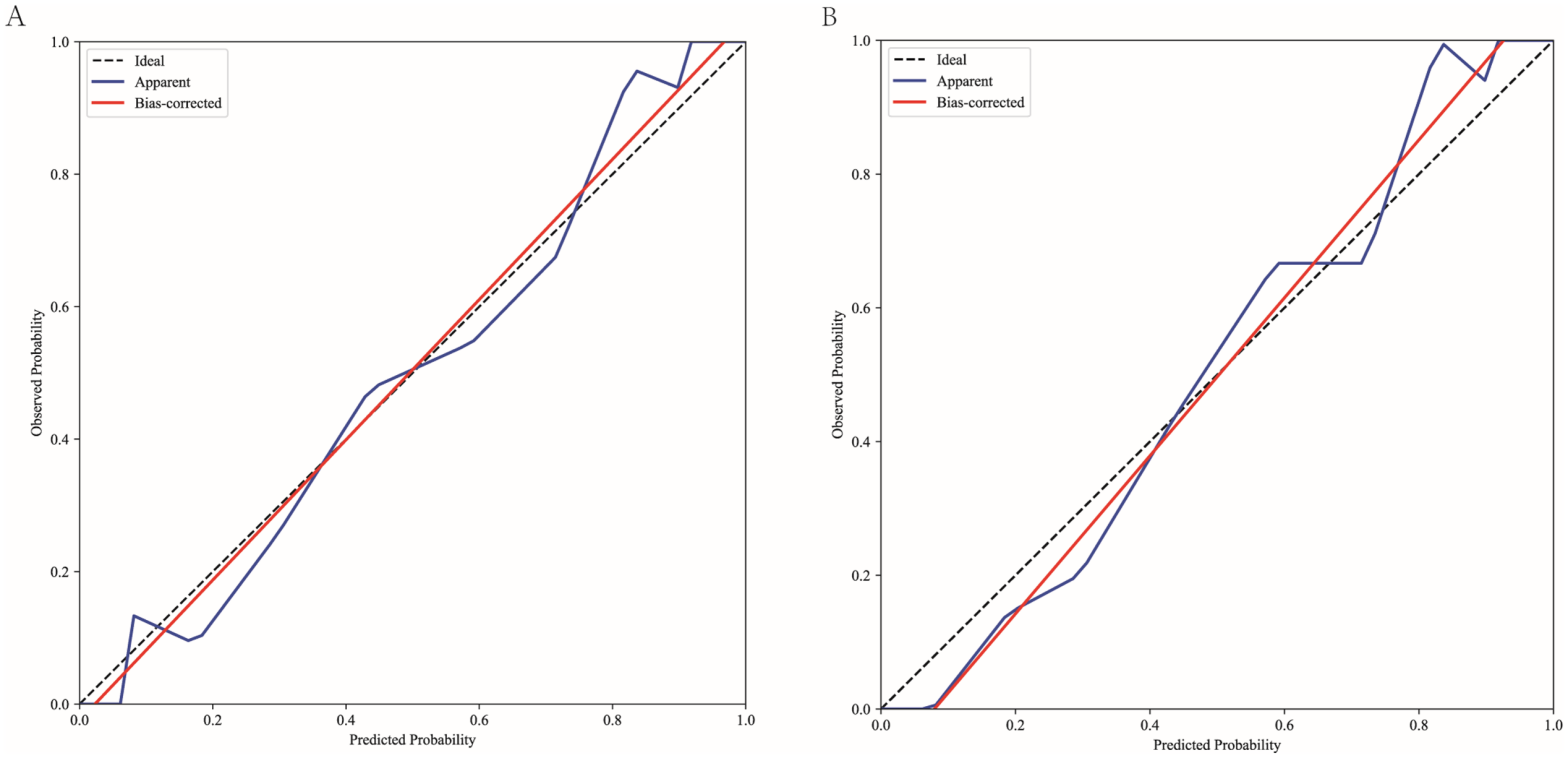
Calibration curves of the nomogram for non-RTW prediction in the cohort. **(A)** In the training cohort, the Hosmer–Lemeshow test yielded χ^2^ = 3.828 (degrees of freedom = 6, *p* = 0.700), with a Brier score of 0.1614, calibration slope of 1.0586, mean absolute error (Eavg) of 0.0567, and R^2^ = 0.9588. **(B)** In the validation cohort, the Hosmer–Lemeshow test yielded χ^2^ = 2.162 (degrees of freedom = 6, *p* = 0.904), with a Brier score of 0.1564, calibration slope of 1.1815, mean absolute error (Eavg) of 0.0658, and R^2^ = 0.9651. The calibration curves suggested good agreement between predicted and observed probabilities in both cohorts. In addition, the non-significant Hosmer–Lemeshow test results (*p* > 0.05) and calibration slopes close to 1.0 supported acceptable model calibration in both the training and validation cohorts.

**Figure 7:**
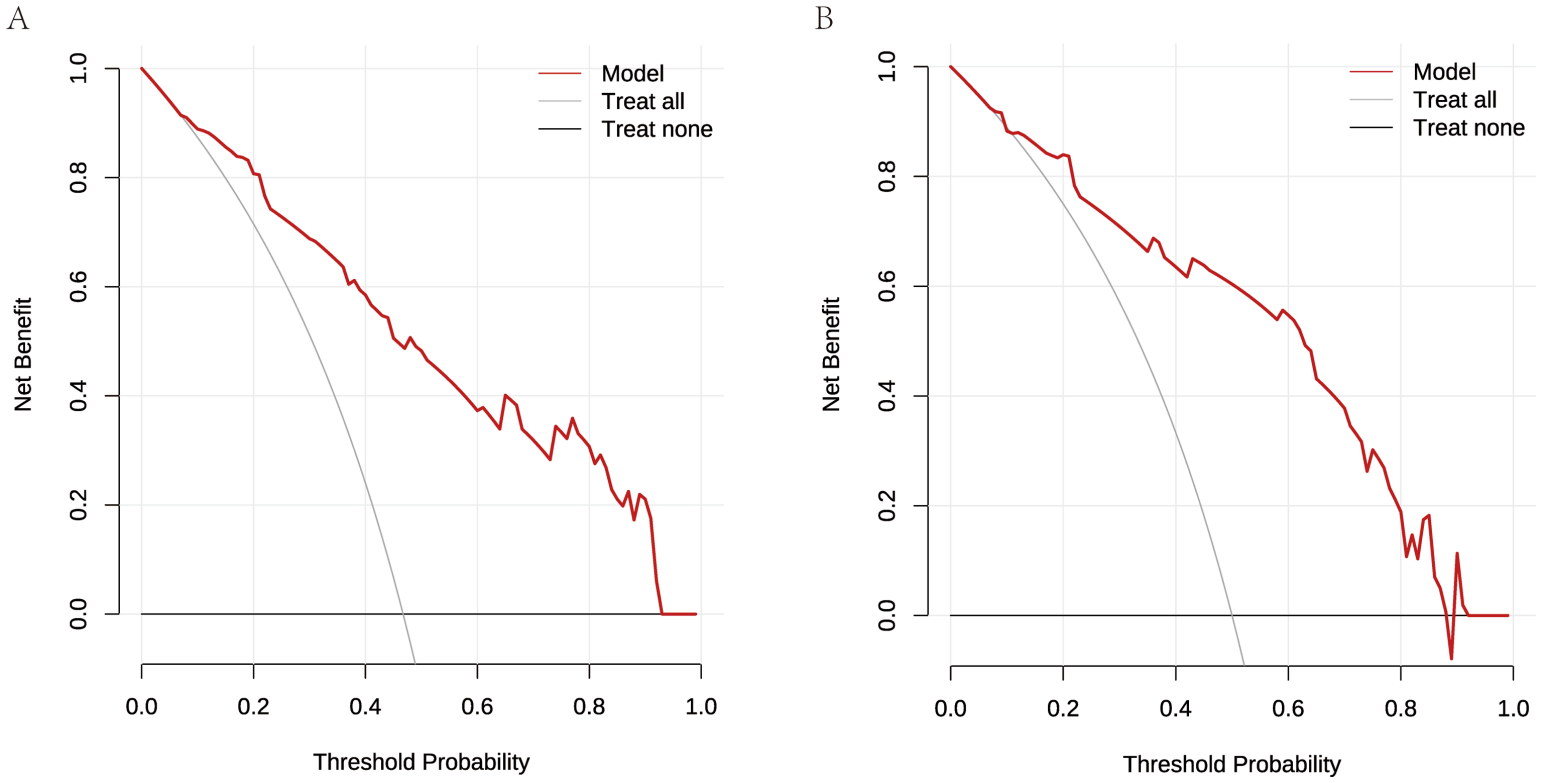
DCA is evaluating the clinical utility of the nomogram. DCA is evaluating the clinical utility of the nomogram. In the training cohort **(A)**, the model exhibited significantly higher clinical net benefit compared with both the “treat-all” and “treat-none” reference strategies across a threshold probability range of 5–92%. In the validation cohort **(B)**, the model consistently outperformed both reference curves within a threshold range of 5–88%. These findings indicate favorable clinical predictive utility in both cohorts, enabling effective identification of high-risk patients with non-RTW who may warrant targeted intervention.

**Figure 8:**
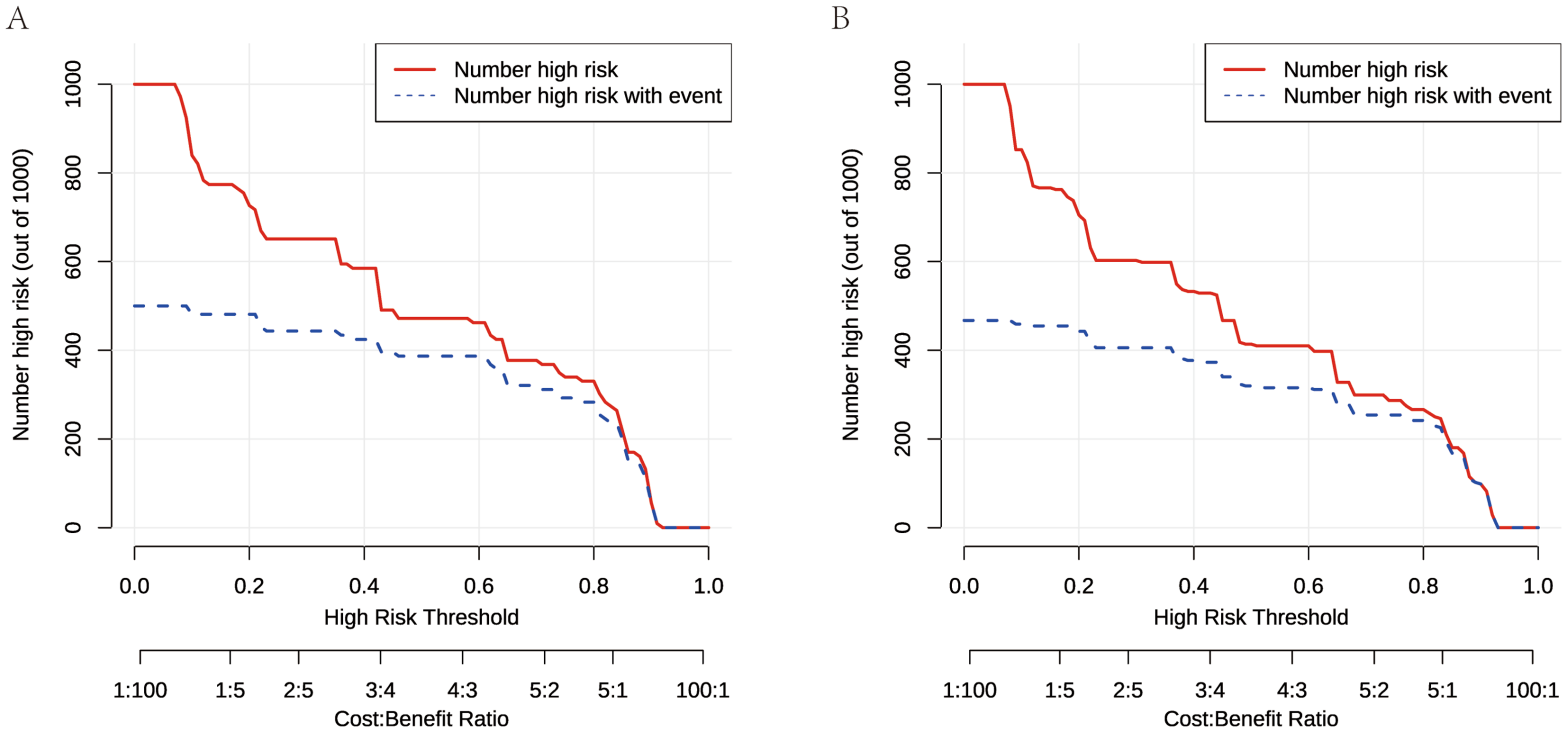
CIC analysis of the nomogram. To evaluate model efficiency under clinical intervention, clinical impact curves (CIC) were constructed for both cohorts **(A,B)**. The red solid line represents high-risk patients; the blue dashed line indicates actual non-RTW cases; the x-axis denotes threshold probability. High-risk patients declined stepwise with increasing threshold. Beyond 0.2, curve convergence indicated improved precision in identifying true events.

Model clinical utility was comprehensively evaluated: the model’s ability to achieve agreement between predicted probabilities and actual non-RTW incidence, as demonstrated by calibration curves (Figures 6A,B) and the Hosmer-Lemeshow test; decision curve analysis demonstrated substantial net clinical benefit across relevant threshold probabilities (Figures 7A,B); additionally, the CIC simulated real-world scenarios and showed high concordance between predicted high-risk patient numbers and observed outcomes (Figures 8A,B). Collectively, these findings confirm that the nomogram possesses reliable clinical utility in predicting postoperative non-RTW following EVT in patients with AIS-LVO.

### 3.8 Temporal confounding effect analysis

**Figure 9:**
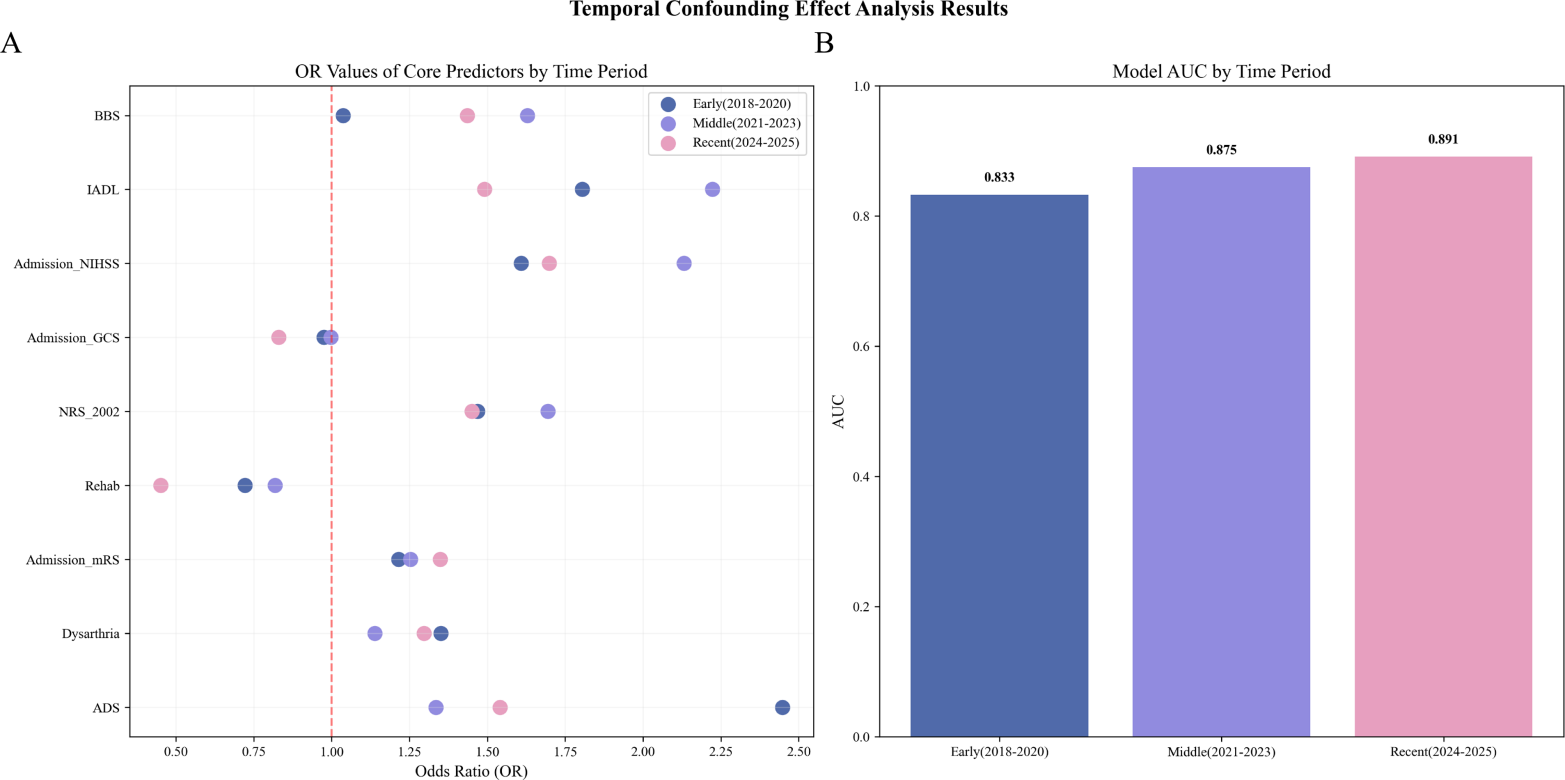
Temporal stability of the predictive model across three clinical eras. **(A)** Odds ratios (ORs) of the six core nomogram predictors for non-return to work in the early (2018–2020, *n* = 86), middle (2021–2023, *n* = 139), and recent (2024–2025, *n* = 125) periods. All predictors maintained directionally consistent effects across eras. **(B)** Discriminative performance (AUC) of the multivariable model within each era, demonstrating stable predictive accuracy over the 7-year study period despite evolving EVT techniques and peri-procedural care protocols.

To evaluate robustness against temporal changes in clinical practice, the cohort was stratified into early (2018–2020, *n* = 86), middle (2021–2023, *n* = 139), and recent (2024–2025, *n* = 125) periods. The nomogram demonstrated consistent discriminative performance across all three eras, with AUCs of 0.833, 0.875, and 0.891, respectively. All six core predictors—admission NIHSS, IADL, BBS, NRS-2002, rehabilitation, and ADS—maintained directionally consistent effects across strata (Figure 9; Supplementary Table S2). For example, admission NIHSS remained a significant risk factor across all eras (ORs ranging from 1.61 to 2.13), as did NRS-2002 (ORs 1.47–1.69) and rehabilitation (ORs 0.45–0.82). Five of six predictors remained statistically significant in each era; BBS showed a directionally consistent association in the early era but did not reach conventional statistical significance (OR = 1.04, 95% CI: 0.83–1.29; *p* = 0.744), likely reflecting limited statistical power in the smallest stratum. Furthermore, when calendar year was incorporated as a continuous covariate into the main multivariable model, it was not significantly associated with non-RTW (OR = 0.96, 95% CI: 0.86–1.07; *p* = 0.439), and the magnitude, direction, and significance of the six core predictors remained materially unchanged (Supplementary Table S3). These findings indicate that the identified predictors are robust to temporal confounding.

### 3.9 Sensitivity analysis

**Figure 10:**
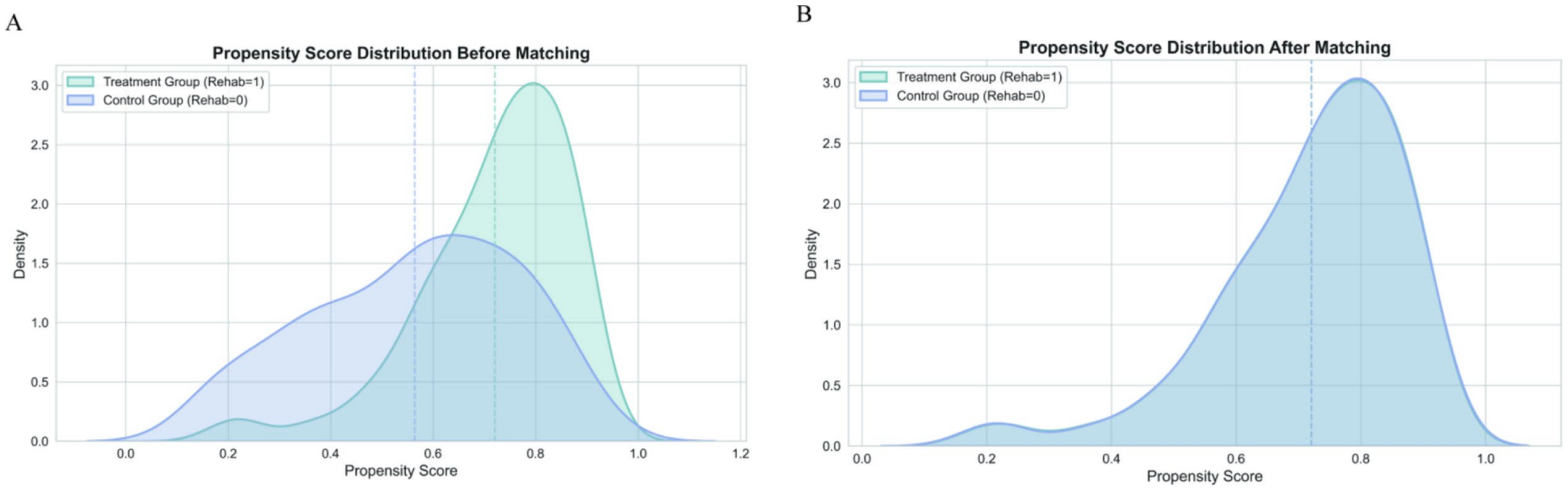
Propensity score distributions before **(A)** and after **(B)** 1:1 nearest-neighbor matching. Before matching, the rehabilitation group (green) showed a higher mean propensity score than the non-rehabilitation group (blue), indicating preferential referral of more severely impaired patients. After matching, the distributions overlapped substantially, supporting adequate covariate balance.

**Figure 11:**
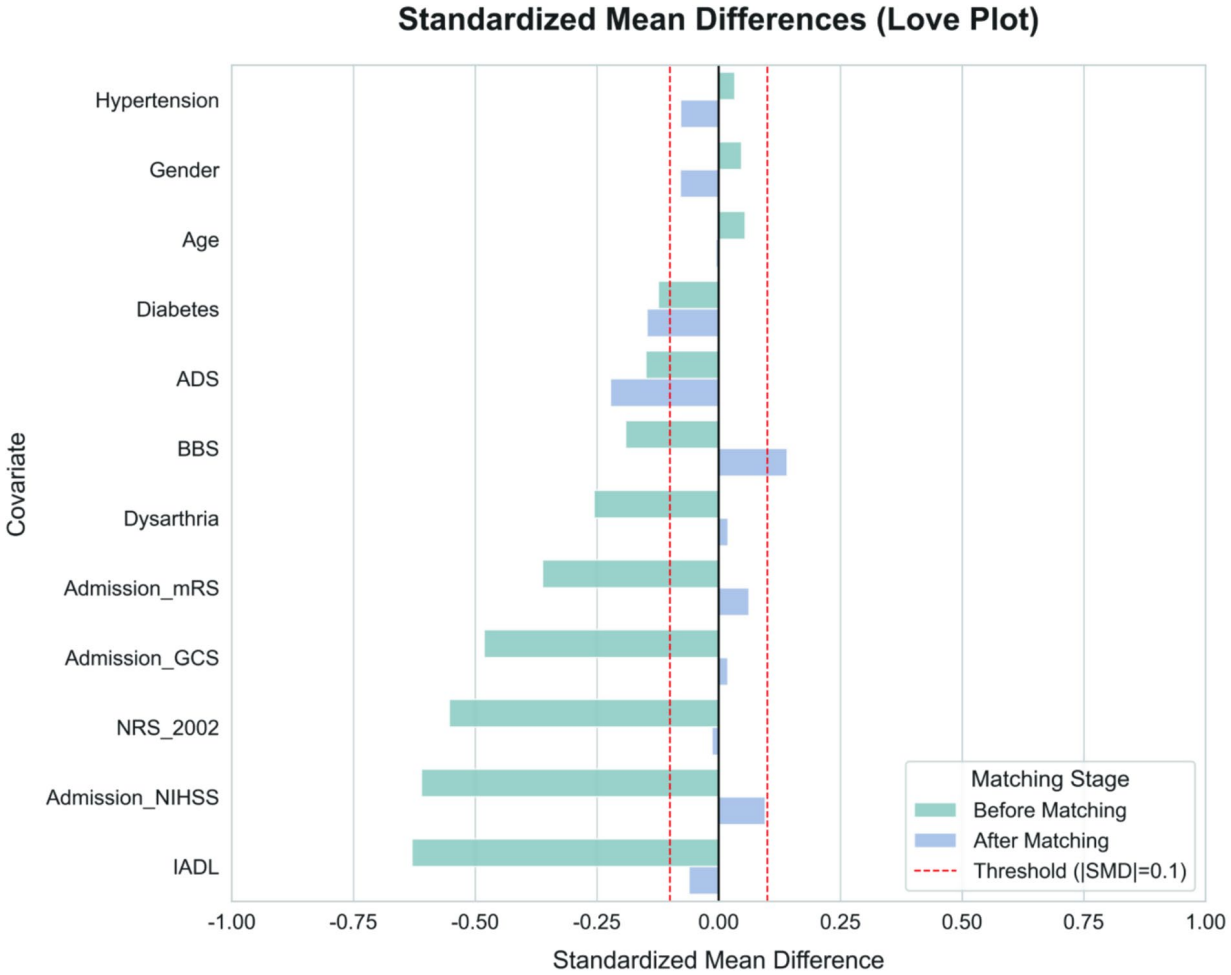
Standardized mean differences (Love plot) for 12 covariates before and after propensity score matching. Red dashed lines indicate the |SMD| = 0.10 threshold. After matching (blue bars), the majority of covariates achieved adequate balance below this threshold, with only minor residual imbalance observed for ADS and BBS, both of which improved substantially compared with pre-matching values.

To address potential confounding by indication, we performed propensity score matching. Figure 10 shows that the propensity score distributions overlapped substantially after 1:1 nearest-neighbor matching. Figure 11 demonstrates that the majority of covariates achieved adequate balance (standardized mean differences <0.10); minor residual imbalance remained for ADS (SMD ≈ −0.15) and BBS (SMD ≈ +0.13), both well below the 0.20 threshold and markedly improved from pre-matching values.

In the matched cohort, rehabilitation remained significantly associated with a lower risk of non-return-to-work. The average treatment effect on the treated (ATT) was −0.1209 (SE = 0.0440, *p* = 0.002), indicating an absolute reduction of approximately 12.1% in the probability of non-return-to-work among treated patients. These findings suggest that the association between rehabilitation and return-to-work outcomes persisted after adjustment for measured confounders.

## 4 Discussion

With the increasing global burden of stroke and the trend toward younger onset (26), occupational functional recovery after EVT for AIS-LVO in young and middle-aged adults has emerged as a major public health concern. In this retrospective study, we found that the rate of non-RTW at 90 days post-EVT among young and middle-aged patients was as high as 47.71%. Although substantial evidence has demonstrated that EVT significantly improves acute neurological outcomes in patients with AIS-LVO, post-stroke occupational reintegration in this population remains suboptimal (12). Tan et al. reported that each young or middle-aged patient who non-RTW results in an average annual indirect socioeconomic loss of approximately RMB 560,000 (8). This not only imposes a substantial financial burden on families and society but also exacerbates labor force shortages from a macro–public health perspective (27).

Absence of post-stroke rehabilitation emerged as an independent predictor of non-RTW following EVT in AIS-LVO patients. In contrast, IADL, admission NIHSS score, nutritional risk (NRS-2002), BBS score, and ADS were independent risk factors. This predictive model transcends the traditional focus on neurological impairment alone by integrating key dimensions such as physical function, nutritional status, psychological factors, and rehabilitative interventions. As such, it aligns closely with the emerging international paradigm of “whole-course, multidimensional prognostic assessment” in stroke care and provides a more comprehensive framework for identifying high-risk individuals in clinical practice (28).

Neurological impairment and physical dysfunction constitute the physiological basis underlying impaired RTW after EVT in young and middle-aged patients with AIS-LVO. A higher admission NIHSS score quantitatively reflects the severity of acute neurological injury across core functional domains, including motor, sensory, language, and visual fields (29). In this study, patients classified as NIHSS grade 2 (5–15 points) and grade 3 (>16 points) exhibited reductions in 90-day RTW rates of 72.38 and 6.28%, respectively, compared with those with grade 1 scores (0–4 points) (*p* < 0.001). Patients with higher NIHSS scores often present with severe hemiplegia, aphasia, or neglect (30), which markedly impair fine motor control, communication efficiency, and spatial judgment, thereby preventing them from meeting the functional demands of their original occupations. These findings indicate that a high admission NIHSS score constitutes a rigid physiological barrier to occupational reintegration and significantly reduces the likelihood of RTW at 90 days.

From a physical function perspective, the IADL scale is a core instrument for assessing independent living and occupational participation (31), encompassing complex activities such as shopping, cooking, financial management, and medication administration. Our findings demonstrate that discharge IADL scores strongly predict RTW outcomes; patients with IADL levels 3–4 (moderate to severe dependence) had significantly lower RTW rates than those with level 1 (full independence). Low IADL scores may reflect impairments in executive function (e.g., planning and organization), leading to a marked decline in work efficiency compared with pre-stroke levels (32). Importantly, neurological impairment and physical dysfunction exert synergistic effects. Motor control deficits associated with high NIHSS scores, combined with balance impairment reflected by low IADL scores, can prevent patients from completing tasks requiring bilateral coordination. Additionally, a subset of patients exhibits a dissociation between neurological and functional recovery: even when NIHSS scores improve (e.g., recovery of language function), persistent fatigue associated with low IADL scores may continue to reduce work efficiency (33). These findings suggest that post-EVT management for young and middle-aged patients should extend beyond neurological recovery alone, emphasizing early involvement of occupational therapy teams to develop rehabilitation programs oriented toward occupational reintegration and coordinated neuro–physical–vocational recovery.

Balance function is a critical determinant of post-stroke functional recovery and plays a pivotal role in occupational reintegration after EVT in young and middle-aged AIS-LVO patients. The BBS, which comprehensively evaluates static balance, dynamic balance, and functional activities, provides a quantitative measure of balance control and indirectly reflects independence in daily living (34). In this study, compared with the High BBS group (score ≥45, low fall risk), the Medium BBS group (score 21–44, moderate fall risk) was identified as an independent predictor of non-RTW (OR = 2.452, *p* = 0.016). Although the association for the Low BBS group (score 0–20, high fall risk) did not reach statistical significance (OR = 2.425, *p* = 0.086), the direction of effect was consistent. These findings suggest that impaired balance function, reflected by lower BBS scores, is associated with less favorable return-to-work outcomes, highlighting the importance of physical stability in occupational reconstruction. Blum L et al. demonstrated a strong positive correlation between BBS and the Barthel Index (34), indicating that balance impairment directly restricts the ability to perform basic activities of daily living (35), thereby limiting occupational participation (36). Even in patients with preserved muscle strength, balance dysfunction increases the risk of falls and disproportionately affects occupations requiring mobility, coordination, or prolonged standing.

Although rehabilitation remained independently associated with a lower risk of non-return-to-work after adjustment for measured confounders, the observational nature of this study precludes causal inference. Experimental studies suggest that rehabilitation-related exercise may facilitate functional recovery through neuroplastic mechanisms. Animal studies have shown that exercise training upregulates brain-derived neurotrophic factor (BDNF) expression in peri-infarct regions, activates BDNF signaling pathways, and enhances synaptic plasticity and neuronal integration (37, 38). Clinical studies have also reported associations between aerobic exercise and improved neurological recovery after stroke (39). Furthermore, earlier rehabilitation initiation has been associated with better functional outcomes, including greater functional independence and improved balance performance (40). These findings provide biological and clinical plausibility for the observed association between rehabilitation and RTW outcomes in our cohort. However, residual confounding and indication bias cannot be completely excluded, and prospective studies are needed to clarify whether rehabilitation exerts a causal effect on RTW after EVT.

Temporal stability. The study spanned a 7-year period during which EVT techniques and peri-procedural care evolved considerably. Time-stratified sensitivity analyses demonstrated consistent effect directions for all six nomogram predictors across three eras, and calendar year showed no significant association with non-RTW when entered as a continuous covariate. These findings suggest that the core determinants of vocational reintegration—neurological injury severity, functional independence, nutritional status, psychological state, and rehabilitation—remain stable predictors despite advances in acute stroke care, and that the nomogram is generalizable across the study period.

Nutritional risk (NRS-2002 score ≥3) (41) and sleep disorders represent novel non-neurological predictors in RTW models for young and middle-aged AIS-LVO patients. Nutritional risk impairs recovery through three primary mechanisms: metabolic dysfunction, impaired neuroplasticity, and accelerated muscle catabolism. Specifically, patients at nutritional risk exhibit a hypercatabolic state characterized by hypoalbuminemia and hypoprealbuminemia, which reflect deficient protein synthesis and impede neural repair (42). Sato H et al. demonstrated that protein malnutrition disrupts the BDNF/TrkB signaling pathway (43), reducing BDNF levels. As a key mediator of neuroplasticity, BDNF expression is positively correlated with recovery of skilled motor function; its deficiency markedly delays fine motor reconstruction (44). In addition, hypoalbuminemia leads to insufficient amino acid substrates, limiting mitochondrial protein synthesis and reducing ATP production (45). Recent studies suggest that reduced ATP synthesis is a central mechanism underlying post-stroke neurological dysfunction and is associated with muscle weakness, atrophy, and loss of motor control (46, 47), thereby impeding rehabilitation and RTW. Notably, young and middle-aged individuals exhibit a daily muscle protein breakdown rate of 2–3% (48). Nutritional risk increases the likelihood of sarcopenia by inhibiting the PI3K/Akt–mTOR pathway and activating FOXO3-mediated proteasomal systems (46, 49), decreasing muscle contraction efficiency, ultimately preventing patients from achieving occupational endurance requirements.

ADS constitute major barriers to RTW through an “emotion–cognition–function” pathway. First, emotional disorders directly impair executive function, attention, and working memory, leading to a significant decline in work capacity (50). Second, anxiety-depressive states are closely associated with post-stroke fatigue, which is one of the core factors hindering RTW. Third, emotional disorders reduce patients’ motivation and adherence to rehabilitation and other treatments (51), thereby forming a vicious cycle of “depression–delayed functional recovery–RTW failure.” The trial conducted by Ntsiea et al. demonstrated that workplace-based interventions incorporating psychological support and fatigue management significantly improved RTW rates (52), highlighting the critical value of psychological interventions in vocational rehabilitation. In addition, neuroimaging and neuropsychological studies have shown that depression is associated with functional abnormalities of the prefrontal cortex, resulting in impaired cognitive control, which further hampers the RTW process (53). Notably, anxiety-depressive state exhibits a bidirectional relationship with malnutrition. Gu et al. reported that malnutrition was significantly associated with an increased risk of post-stroke depression, and that the reduction in depressive risk occurred more slowly among malnourished patients (54). Given that young and middle-aged stroke survivors bear dual family and social role pressures, they are particularly vulnerable to a negative feedback loop of “anxiety/depression–delayed rehabilitation–RTW failure.”

Therefore, we recommend the establishment of a combined “nutrition–psychological” screening pathway. For patients with an NRS-2002 score≥3, early initiation of nutritional intervention is warranted, while those with comorbid anxiety and depressive state should receive timely cognitive behavioral therapy. By disrupting the vicious cycle of “malnutrition–emotional disturbance–functional impairment,” RTW outcomes may be substantially optimized.

## 5 Conclusion

We developed and validated a prognostic nomogram (AUC = 0.858) using six independent predictors (admission NIHSS, IADL, Berg Balance Scale, rehabilitation, nutritional risk, ADS) to predict non-RTW risk in young and middle-aged AIS-LVO patients after EVT. A key strength is its focus on functional recovery rather than routine laboratory markers, which often normalize in non-RTW patients, making functional assessment key for occupational reintegration.

Clinically, this nomogram helps early identify high-risk patients for timely multidisciplinary intervention and guides individualized management (e.g., nutritional support, intensified rehabilitation).

Our results highlight that post-EVT management for young and middle-aged stroke survivors should combine neurological recovery with nutritional screening, psychological intervention, and vocation-oriented rehabilitation to improve long-term function, facilitate return to work, and enhance quality of life.

## 6 Limitations

Several limitations should be acknowledged. First, although temporal sensitivity analyses demonstrated stable model performance across different enrollment periods and no significant linear era effect was observed (*p* = 0.439), the study spanned a period during which EVT techniques, post-procedural care, and rehabilitation practices evolved. Therefore, residual temporal confounding cannot be completely excluded.

Second, this was a single-center retrospective study, and the 7:3 random split used for model development and validation represents an internal random-split validation rather than true external validation. Although similar performance was observed in the training and validation cohorts, the shared institutional setting, clinical workflows, and treatment practices may have contributed to optimistic estimates of model performance. Consequently, the generalizability of the nomogram to independent populations remains to be established through external validation.

Third, the sample size was modest, particularly in the validation cohort, which may have limited the precision of performance estimates and calibration assessment. Larger studies are warranted to further evaluate model robustness.

Fourth, several potentially important occupational, psychosocial, and treatment-related factors, including prestroke job demands, workplace support, socioeconomic status, educational attainment, rehabilitation intensity, patient motivation, and family support, were unavailable in this retrospective dataset and therefore could not be incorporated into the prediction model. Consequently, residual confounding cannot be completely excluded, particularly in the observed association between post-stroke rehabilitation and RTW outcomes.

Fifth, the operational definition of return to work, including the 70% work-intensity threshold and minimum workload of 10 h per week, was based on pragmatic considerations and has not been specifically validated in patients with AIS-LVO undergoing EVT.

Finally, follow-up was limited to 90 days after EVT. Although this time point is clinically relevant for early recovery, it may not fully capture longer-term vocational outcomes, which are commonly assessed at 6–12 months after stroke. Future multicenter prospective studies with extended follow-up are needed to externally validate the model and evaluate long-term return-to-work trajectories.

## Data Availability

The original contributions presented in the study are included in the article/Supplementary material, further inquiries can be directed to the corresponding authors.
