# Supplementary material for: A clinically applicable nomogram predicting non-return to work in young and middle-aged patients with acute large vessel occlusion stroke: integrating neurological function and psychosocial factors for personalized rehabilitation
Source: Front Neurol. 2026 Jun 24;17:1837086. doi: 10.3389/fneur.2026.1837086 (PMC13341439; doi:10.3389/fneur.2026.1837086)
Supplement: Supplementary file 1 [file Table_1.DOCX]

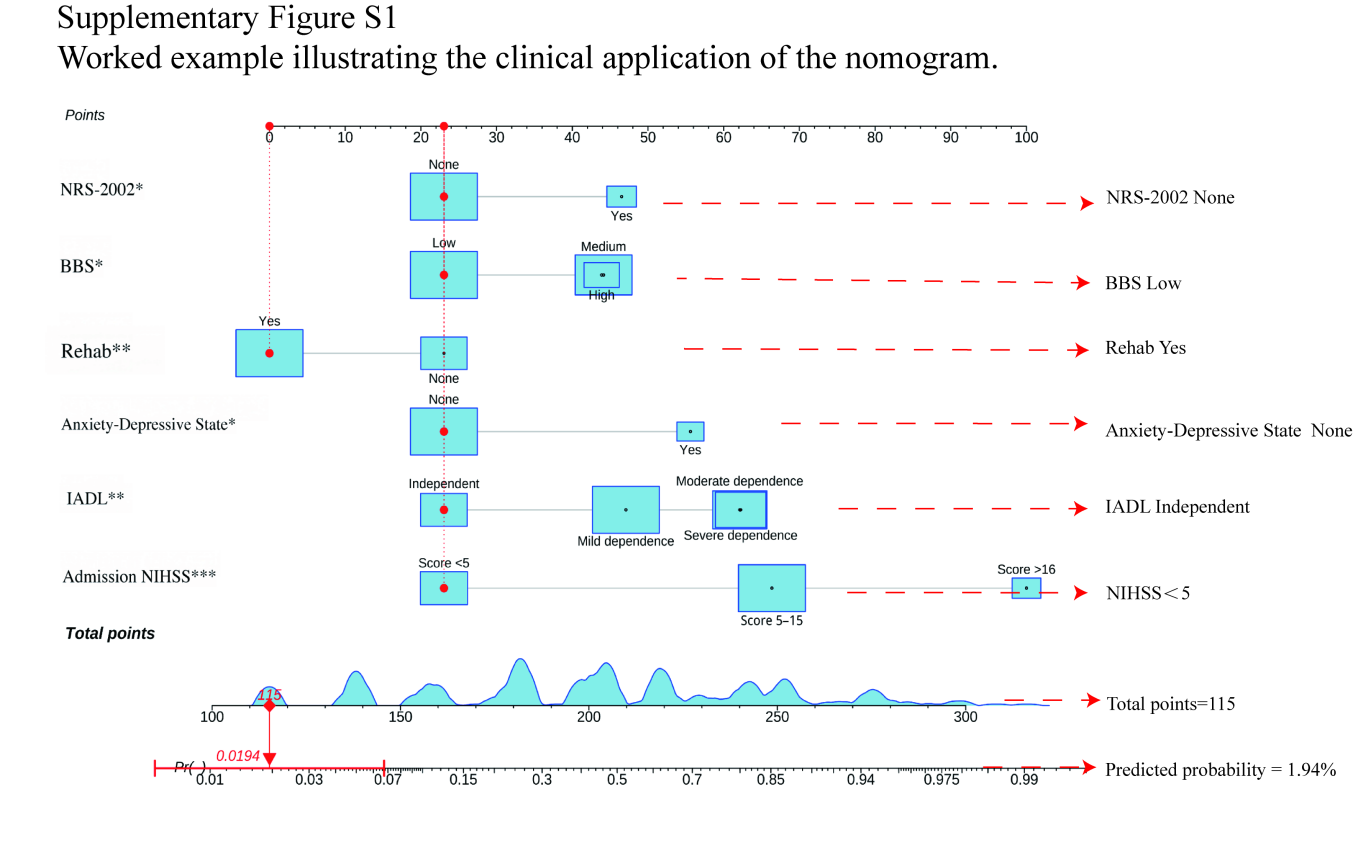


Supplementary Figure S1. Worked example illustrating the clinical application of the nomogram. A patient with admission NIHSS < 5, independent IADL status, no nutritional risk (NRS-2002 < 3), no anxiety-depressive state, receipt of post-stroke rehabilitation, and a low-risk BBS category accumulates approximately 115 total points. According to the nomogram, this corresponds to an estimated 90-day probability of non-return-to-work of 1.94%.
