# Supplementary material for: A clinically applicable nomogram predicting non-return to work in young and middle-aged patients with acute large vessel occlusion stroke: integrating neurological function and psychosocial factors for personalized rehabilitation
Source: Front Neurol. 2026 Jun 24;17:1837086. doi: 10.3389/fneur.2026.1837086 (PMC13341439; doi:10.3389/fneur.2026.1837086)
Supplement: Supplementary file 2 [file Table_2.DOCX]

**Table S1. Variable-specific missing data counts and missingness rates used for multiple imputation**

| **Variable Category** | **Variables** | **Missing Count (n)** | **Missingness Rate (%)** |
| --- | --- | --- | --- |
| **Demographics** | Age | 0 | 0.0 |
|  | Sex | 0 | 0.0 |
|  | Marital status | 2 | 0.6 |
|  | Income level | 5 | 1.4 |
| **Vascular Risk Factors** | Hypertension | 0 | 0.0 |
|  | Diabetes | 0 | 0.0 |
|  | Coronary disease | 3 | 0.9 |
|  | Smoking | 4 | 1.1 |
| **Stroke Etiology** | TOAST classification | 0 | 0.0 |
| **Neurological & Functional** | Admission NIHSS | 5 | 1.4 |
|  | Admission GCS | 5 | 1.4 |
|  | Admission mRS | 6 | 1.7 |
|  | IADL (Lawton) | 14 | 4.0 |
|  | BBS | 17 | 4.9 |
|  | Dysarthria | 8 | 2.3 |
|  | Sensory deficits (Paresthesia) | 10 | 2.9 |
| **Psychological & Sleep** | ADS (HAMA/HAMD) | 18 | 5.1 |
|  | Sleep quality (PSQI) | 20 | 5.7 |
| **Nutritional** | Nutritional risk (NRS-2002) | 12 | 3.4 |
| **Procedural** | EVT duration | 4 | 1.1 |
| **Postoperative Variables** | HbA1c | 15 | 4.3 |
|  | Serum creatinine | 8 | 2.3 |
|  | LVEF | 13 | 3.7 |
|  | Fazekas grade | 14 | 4.0 |
|  | PNI | 16 | 4.6 |
|  | AI | 20 | 5.7 |
| **Treatment** | Post-stroke rehabilitation | 0 | 0.0 |
