# Supplementary material for: A clinically applicable nomogram predicting non-return to work in young and middle-aged patients with acute large vessel occlusion stroke: integrating neurological function and psychosocial factors for personalized rehabilitation
Source: Front Neurol. 2026 Jun 24;17:1837086. doi: 10.3389/fneur.2026.1837086 (PMC13341439; doi:10.3389/fneur.2026.1837086)
Supplement: Supplementary file 3 [file Table_3.DOCX]

**Table S2. Time-stratified multivariable logistic regression for non-return to work across three clinical eras**

| **Variables** | **Early (2018–2020) n = 86** | **Middle (2021–2023) n = 139** | **Recent (2024–2025) n = 125** |
| --- | --- | --- | --- |
|  | OR (95% CI); P | OR (95% CI); P | OR (95% CI); P |
| ADS | 2.45 (1.96–3.05); <0.001 | 1.34 (1.12–1.60); 0.002 | 1.54 (1.28–1.85); <0.001 |
| Rehab | 0.72 (0.58–0.90); 0.004 | 0.82 (0.68–0.99); 0.037 | 0.45 (0.37–0.55); <0.001 |
| NRS-2002 | 1.47 (1.15–1.87); 0.002 | 1.69 (1.41–2.05); <0.001 | 1.45 (1.18–1.79); <0.001 |
| Admission NIHSS | 1.61 (1.13–2.29); 0.008 | 2.13 (1.57–2.90); <0.001 | 1.70 (1.25–2.31); <0.001 |
| BBS | 1.04 (0.83–1.29); 0.744 | 1.63 (1.36–1.95); <0.001 | 1.44 (1.19–1.73); <0.001 |
| IADL | 1.81 (1.39–2.35); <0.001 | 2.22 (1.83–2.70); <0.001 | 1.49 (1.22–1.82); <0.001 |
| Model performance |  |  |  |
| AUC (95% CI) | 0.833 | 0.875 | 0.891 |

**Note:** **OR,** odds ratio; **CI,** confidence interval; **AUC,** area under the receiver operating characteristic curve. Bold variables represent the six predictors retained in the final nomogram. The BBS did not reach conventional statistical significance in the early era (P = 0.744), likely reflecting limited statistical power in the smallest stratum (n = 86).
