# Supplementary material for: A clinically applicable nomogram predicting non-return to work in young and middle-aged patients with acute large vessel occlusion stroke: integrating neurological function and psychosocial factors for personalized rehabilitation
Source: Front Neurol. 2026 Jun 24;17:1837086. doi: 10.3389/fneur.2026.1837086 (PMC13341439; doi:10.3389/fneur.2026.1837086)
Supplement: Supplementary file 4 [file Table_4.DOCX]

**Table S3. Sensitivity analysis with calendar year as a continuous covariate in the main multivariable model**

| **Variables** | **Coefficient** | **OR (95% CI)** | **P-value** |
| --- | --- | --- | --- |
| ADS | 0.538 | 1.71 (1.44–1.80) | <0.001 |
| Rehab | −0.489 | 0.61 (0.57–0.72) | <0.001 |
| NRS-2002 | 0.553 | 1.74 (1.50–2.01) | <0.001 |
| Admission NIHSS | 0.608 | 1.84 (1.46–2.07) | <0.001 |
| BBS | 0.322 | 1.38 (1.25–1.56) | <0.001 |
| IADL | 0.700 | 2.01 (1.58–2.10) | <0.001 |
| Year | −0.043 | 0.96 (0.86–1.07) | 0.439 |

**Note:** Year was entered as a continuous variable (range: 2018–2025) to assess linear temporal trends. The non-significant association (P = 0.439, 95% CI crossing 1.0) indicates no meaningful linear era effect on non-RTW.
